# Design and applications of self-assembled polypeptide matrices in wound healing

**DOI:** 10.3389/fbioe.2025.1646622

**Published:** 2025-08-11

**Authors:** Mélanie Côté-Cyr, Steve Bourgault

**Affiliations:** ^1^ Department of Chemistry, Université du Québec à Montréal, Montreal, QC, Canada; ^2^ Quebec Network for Research on Protein Function, Engineering and Applications (PROTEO), Montreal, QC, Canada

**Keywords:** proteins, peptides, self-assembly, hydrogels, matrices, wound healing, cell adhesion, extracellular matrix

## Abstract

With an estimated prevalence of over two cases per 1,000 patients, chronic wounds represent a massive burden on healthcare systems around the globe. Such wounds often lead to major complications, including amputations, that greatly affect the living conditions of patients. Typical therapeutic approaches include skin grafts and topical application of therapeutic molecules such as growth factors. Current limitations of grafts include the availability of healthy tissues and risks of rejection, while the efficiency of therapeutic molecules is limited by their short half-life in the wound environment. Interestingly, porous matrices such as hydrogels have emerged as promising materials by acting simultaneously as a scaffold for skin cell proliferation and as a delivery system for therapeutic molecules, protecting them from degradation and/or elimination. Self-assembling polypeptides have revealed interesting properties for the fabrication of such materials, notably their ability to mimic the extracellular matrix of the skin, tunable mechanical properties and ease of conjugation to bioactive sequences. In this context, the present review aims at highlighting the diversity of self-assembled protein and peptide-based matrices, natural and synthetic, that have been evaluated as wound healing scaffolds. After briefly describing the most common bioactive protein sequences used within these matrices, examples of nature-inspired and synthetic self-assembled proteinaceous matrices studied for wound healing will be presented. Finally, strategies for modulating the mechanical properties of the hydrogels are discussed. Despite the number of studies published on the subject, the expanding number of self-assembling protein sequences and the constantly improving strategies for modulating the mechanical properties of resulting matrices should further drive the development of improved protein-based hydrogels for wound healing.

## Introduction

Skin wounds and specifically chronic wounds represent a significant burden on healthcare systems across the world (Nussbaum et al., 2018; Martinengo et al., 2019). Conditions such as diabetes, high alcohol consumption, aging and other chronic diseases increase the risk of developing chronic wounds (Guo and Dipietro, 2010). Complications associated with these wounds result in substantial medical costs as well as high levels of stress, pain, and discomfort in patients (Nussbaum et al., 2018; Sen, 2019). Development of efficient wound healing technologies and materials therefore remains a prominent public health issue. Although several approaches have been developed to treat difficult wounds, each of them shows some limitations. For example, autografts, which have long been considered an optimal approach for wound treatment, are limited to small size wounds and by the availability of healthy tissue in the patient (Koller, 2005; Simman and Phavixay, 2011; Carter and Holmes, 2016). Allogenic skin grafts are also limited by availability, as well as risk of rejection (Koller, 2005; Benichou et al., 2011). For these reasons, efforts have recently focused on the engineering of cell-free hydrogels and matrices as wound healing technologies. These materials can act not only as a scaffold for cell attachment and proliferation, but also as a delivery system for sustained release of bioactive molecules.

The optimal matrices for tissue regeneration should be biocompatible and easy to functionalize, or to load, with bioactive molecules. Ideal properties of these matrices also include immunocompatibility, biodegradability and mechanical properties adapted for cell growth within the scaffold (Fan et al., 2021). Over the years, many synthetic polymers have been investigated as hydrogel scaffolds for wound healing, including polyethylene glycol (PEG) and poly lactic-co-glycolic acid (PLGA) (Chereddy et al., 2016; Chen et al., 2018; Fan et al., 2021). Natural polymers such as polysaccharides and proteins have also been extensively studied as building blocks for wound healing matrices due to their inherent biocompatibility, degradability, viscoelasticity and high degree of hydration (Fan et al., 2021). Specifically, a wide range of self-assembling proteins and peptides have been harnessed for tissue regeneration. Protein-based materials are usually porous and highly hydrated, allowing cell penetration and proliferation, and efficiently mimic the native extracellular matrix (ECM) in the skin (Biggs et al., 2020). Self-assembly proteins usually form nanoparticles or fibrils, some of which can also form higher order assemblies, resulting in attractive materials for wound healing, such as fibre network matrices and hydrogels (Das and Das, 2021). Proteins are also easy to functionalize with bioactive moieties, and the mechanical properties of the resulting self-assembled material can be tailored to their final application using protein engineering and crosslinking strategies.

The variety of available and ever-expanding natural and designed protein sequences with unique biological and self-assembling properties has resulted in development of numerous protein-based scaffolds with applications in regenerative medicine and tissue engineering. While some recent reviews have detailed the use of specific self-assembled protein and protein families in wound healing (Whelan et al., 2014; Rodríguez-Cabello et al., 2018; Chouhan and Mandal, 2020; Naomi et al., 2021; Sharma et al., 2022), reviews featuring a wide variety of self-assembly proteins often also encompass a wide range of biomedical applications. This review article therefore aims to report exclusively on self-assembled protein-based matrices, natural and synthetic, studied in a wound healing context, and to detail their properties in relation to this specific application. Details on bioactive moieties used in wound healing, functionalization of protein matrices and crosslinking strategies are also discussed.

## Bioactive protein sequences

In addition to acting as scaffolds for cell proliferation, protein matrices are easy to functionalize and/or to load with bioactive moieties adapted to different applications. Although topical application of growth factors, antimicrobials and other therapeutic molecules to the wound site has been proven to improve wound healing, the efficacy of this strategy is limited by their short half-life in the wound environment, which is highly concentrated in proteases (Yamakawa and Hayashida, 2019). Fortunately, functionalization and loading of bioactive molecules within protein scaffolds has been shown to extend their half-life and enable sustained release, consequently reducing the concentration of molecules needed to maintain the desired biological effect over a prolonged period (Nurkesh et al., 2020). This first section provides a concise overview of wound healing stages and of some of the most common bioactive molecules used in protein-based wound healing matrices. For more details on the subject, the readers are encouraged to consult the comprehensive reviews of Hosoyama and Ligoiro (Hosoyama et al., 2019; Ligorio and Mata, 2023).

### Stages of wound healing

**FIGURE 1:**
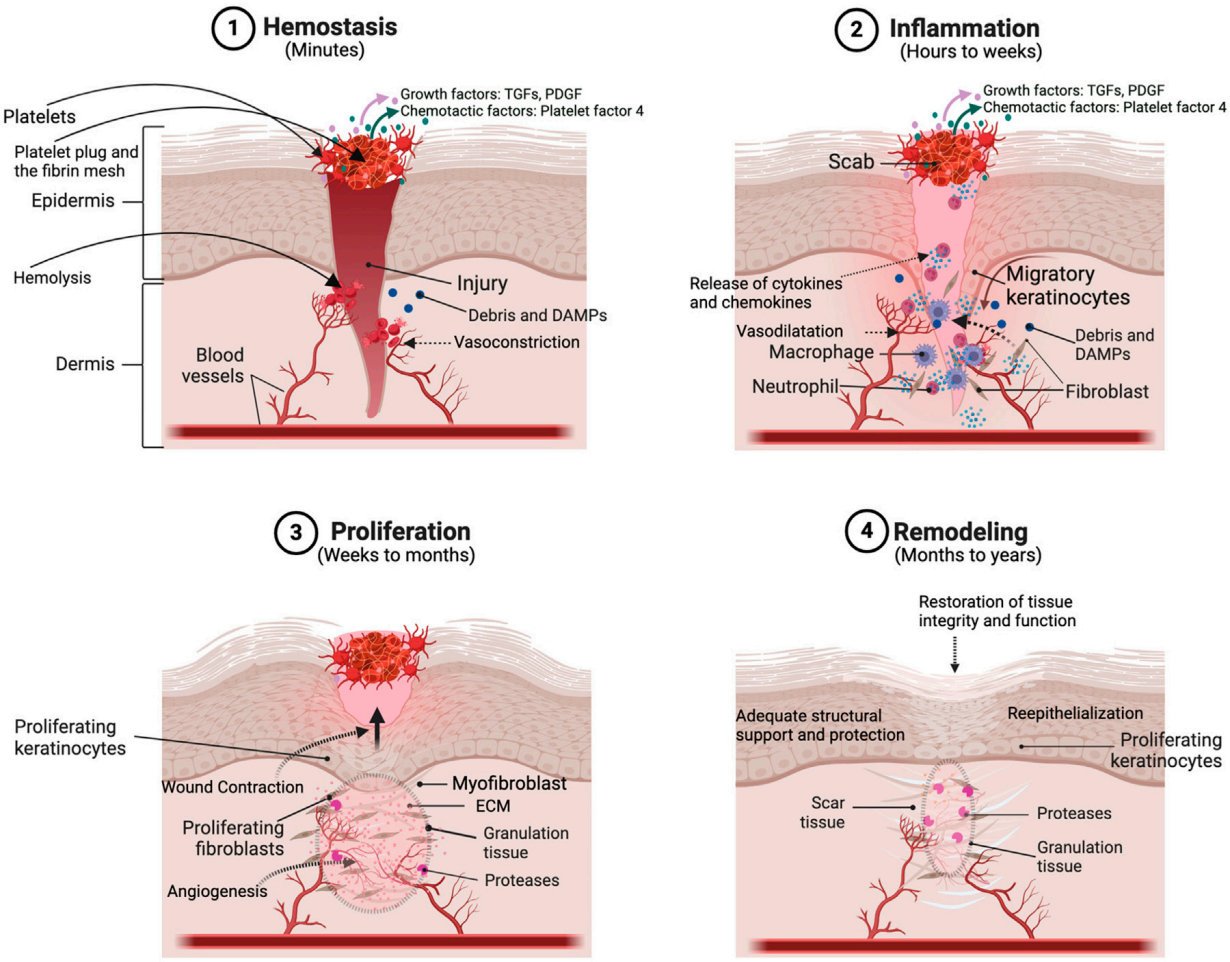
Schema representing the different stages and the principal actors of wound healing. Reprinted with permission from Choudhary et al. (2024).

Full-thickness skin wounds usually heal in four stages. The first stage, hemostasis, takes place during the first minutes following injury and allows bleeding to stop (Figure 1). It then leaves place to the inflammatory phase during which macrophages, neutrophils and other immune cells infiltrate the wound site to eliminate pathogens and remove damaged tissues. Production of cytokines and growth factors by these cells during the late inflammatory phase also allows transition to the proliferative phase, during which mesenchymal and epidermal stem cells differentiate and other cells, including fibroblasts, keratinocytes, and endothelial cells, proliferate. Fibroblasts also produce collagen and other constituents of the ECM. These processes allow angiogenesis (*i.e.*, blood vessel formation) and formation of granulation tissue, which is rich in inflammatory cells, fibroblasts, and new blood vessels. The final phase, remodeling, lasts months to years and allows re-organization of the ECM, maturation of capillaries and recovery of the skin’s initial properties (Guo and Dipietro, 2010; Martin and Nunan, 2015).

Most conditions associated with chronic wounds affect the transition from the inflammatory phase to the proliferative phase (Guo and Dipietro, 2010; Martin and Nunan, 2015). This chronic inflammatory state also results in an increased number of infections in patients. Therefore, antimicrobials and factors targeting the late inflammatory and the proliferative phases of the process (*e.g.,* growth factors and adhesion sequences) are the most widely used bioactive molecules in wound healing. Although full-length proteins could be used, utilization of shorter biomimetic peptide sequences facilitates production and conjugation to protein matrices.

### Cell-adhesion sequences

Cell adhesion to the matrix is crucial during the proliferation phase of wound healing. The ECM in the healthy skin usually provides a support for fibroblasts and endothelial cells. Therefore, most cell adhesion sequences used in biomaterials are derived from ECM proteins such as fibronectin and laminin. The most studied cell-adhesion motif is the RGD tripeptide sequence derived from fibronectin (Table 1). This motif, also present in other ECM proteins (e.g., laminin, vitronectin …), is known as a binding motif for many integrins, including α_5_β_1_, α_5_β_3_ and α_4_β_1_, which are present at the surface of keratinocytes, fibroblasts, and endothelial cells, allowing their attachment and spreading (Dalton et al., 1992; Ruoslahti, 1996; Di Russo et al., 2021). Within a wound healing matrix, RGD and similar sequences (*i.e.*, RGDSP) allow attachment and proliferation of keratinocytes and fibroblasts (Asakura and Naito, 2023) as well as blood vessel formation by endothelial cells (Hersel et al., 2003; Sarangthem et al., 2022). Another common adhesion sequence derived from fibronectin is the PHSRN pentapeptide. This short five-residue sequence interacts with integrins in a similar way to the RGD tripeptide, promoting keratinocyte and fibroblast adhesion to the matrix, spreading and proliferation (Livant et al., 2000; Feng and Mrksich, 2004; Zeng et al., 2009). It also acts in a synergistic manner with RGD sequences and is often used in combination with them in biomaterials (Feng and Mrksich, 2004; Mardilovich et al., 2006; Shroff et al., 2010).

**TABLE 1:** Cell adhesion sequences and growth factors used in wound healing biomaterials.

Type	Protein	Peptide sequence(s)	Biological activities	References
Cell adhesion sequence	Fibronectin	RGD	Adhesion, spreading and proliferation (integrin binding) of fibroblasts, keratinocytes, and endothelial cells, angiogenesis, promotes ECM deposition	Ruoslahti (1996), Massia and Stark (2001), Hersel et al. (2003)
		PHSRN	Adhesion, spreading and proliferation (integrin binding), synergy with RGD, angiogenesis	Livant et al. (2000), Feng and Mrksich (2004), Zeng et al. (2009)
	Laminin	IKVAV	Adhesion and proliferation of MSCs and endothelial cells, angiogenesis, synergy with RGD	Tashiro et al. (1989), Grant et al. (1992), MALINDA et al. (1999), Jung et al. (2011)
		YIGSR	Adhesion and proliferation of MSCs and endothelial cells, angiogenesis	Tashiro et al. (1989), Grant et al. (1992)
		RYVVLPR	Adhesion of keratinocytes and endothelial cells, angiogenesis	Skubitz et al. (1990), Wilke and Skubitz (1991)
Growth factor	VEGF	KLTWQELYQLKYKGI	Proliferation and migration of endothelial cells and keratinocytes, angiogenesis, recruitment of macrophages and precursor cells (FGF receptors activation)	Leung et al. (1989), Galiano et al. (2004), Wilgus et al. (2005), Muramatsu et al. (2010), Finetti et al. (2012)
	EGF	sh-oligopeptide-1	Proliferation and migration of fibroblasts, keratinocytes and endothelial cells, angiogenesis, promotes collagen and ECM deposition	Laato et al. (1987), Wenczak et al. (1992), Bodnar, 2013; Kim et al. (2015)
	FGF-2/b-FGF	YRSRKYSSWYVALKR	Proliferation and differentiation keratinocytes, fibroblasts and endothelial cells, angiogenesis, collagen deposition	Baird et al. (1988), Hebda et al. (1990), Koike et al. (2020)
	FGF1/a-FGF	SKKHAEKNWFVGLKKN and WFVGLKKNGSSKRGPRT	Proliferation and migration of fibroblasts, keratinocytes and endothelial cells, angiogenesis	Thomas et al. (1985), Shipley et al. (1989), Oyama Jr. et al. (1996), Hagerott et al. (2020)
	FGF-7/KGF	KELILENHYNTYA	Proliferation and migration of keratinocytes	Tsuboi et al. (1993), Choudhury et al. (2024), Kim et al. (2025)
	SDF-1/CXCL12	SDF-1-mimic peptide (LSYKCGCKFGGGFRCPCRYSL-NH2)	Chemotaxis, migration and proliferation of epidermal stem cells, endothelial progenitor cells and leukocytes, angiogenesis	Lau and Wang (2011), Guo et al. (2015), Janssens et al. (2018), Kim et al. (2023)

Abbreviations: ECM, extracellular matrix; MSCs, mesenchymal stem cells; VEGF, vascular endothelial growth factor; EGF, epidermal growth factor; FGF, fibroblast growth factor; KGF, keratinocyte growth factor; SDF-1, stromal cell-derived factor-1.

Laminin, another ECM protein, also constitutes a well-known source of cell adhesion motifs. The most common laminin-inspired adhesion sequence is the IKVAV pentapeptide (Table 1). The YIGSR pentapeptide motif, also derived from laminin, can be used as an alternative to IKVAV. Both peptides are known to promote adhesion and proliferation of mesenchymal stem cells (MSC) and endothelial cells through interaction with integrins. In a skin wound context, both peptide factors promote angiogenesis (Tashiro et al., 1989; Grant et al., 1992; MALINDA et al., 1999; Frith et al., 2012). Additionally, IKVAV has been shown to have a synergistic effect with RGD on endothelial cell growth (Jung et al., 2011). The RYVVLPR peptide, is also a laminin-derived adhesion sequence used in biomaterials to promote angiogenesis. Besides, it has been shown to stimulate keratinocyte adhesion (Wilke and Skubitz, 1991). Although mostly used for other tissue engineering applications such as neural regeneration, these laminin-derived sequences are sometimes used in skin wound healing (Genové et al., 2005; Frith et al., 2012; Ligorio and Mata, 2023).

### Growth factors

As previously stated, for problematic wounds, transition from the inflammatory to the proliferative stage of healing is hindered. Such wounds therefore show poor re-epithelialization and re-vascularization. For this reason, addition of growth factors to wound healing biomaterials is a common strategy to address this issue.

Vascular endothelial growth factor A (VEGF-A), commonly referred to as VEGF, is the most abundant proangiogenic factor in the skin and induces neovascularization in wounds (Leung et al., 1989). However, in chronic wounds, VEGF levels are usually lower than expected (Lauer et al., 2000). VEGF has been shown to induce recruitment of macrophages and endothelial precursor cells during the inflammatory phase of wound healing (Galiano et al., 2004; Muramatsu et al., 2010). Its angiogenic activity also allows better oxygenation of the wound environment, which supports the formation of granulation tissue (Romano Di Peppe et al., 2002; Galeano et al., 2003). Some studies also demonstrated the ability of VEGF to stimulate proliferation and migration of keratinocytes (Wilgus et al., 2005; Brem et al., 2009). Although VEGF has been encapsulated and bound to polymeric biomaterials before (Ehrbar et al., 2004; Anderson et al., 2011), its molecular size (45 kDa) represents a limitation for conjugation to self-assembled protein materials and is not compatible with chemical peptide synthesis. Fortunately, a VEGF-mimetic peptide, QK (ac-KLTWQELYQLKYKGI-NH2), has been developed and shows similar angiogenic activity to native VEGF (Table 1) (Finetti et al., 2012). This 15-mer peptide is relatively simple to synthesize chemically and can be easily functionalized to proteinaceous biomaterials (Liu et al., 2012; Kim and Liu, 2016).

Epidermal growth factor (EGF) is another prominent factor enhancing proliferation and migration of fibroblasts (Laato et al., 1987; Kim et al., 2015), keratinocytes (Ryu et al., 2009) and endothelial cells (Wenczak et al., 1992; Bodnar, 2013). Topical application of human recombinant EGF has been reported to accelerate wound healing by promoting re-epithelialization and angiogenesis (Yang et al., 2016). Similarly to VEGF, EGF has been encapsulated and bound to polymeric materials for wound healing. Its size (6.4 kDa), although not as important as VEGF, still makes this growth factor inaccessible by peptide synthesis (Table 1). One EGF-mimicking peptide, the synthetic Human oligopeptide-1 (sh-oligopeptide-1), has been reported in the literature, but little evidence supports its wound-healing properties (Martínez-Carpio, 2023).

Members of the fibroblast growth factor (FGF) family, which comprises 22 growth factors, are also playing key roles in many wound healing processes (Prudovsky, 2021). For instance, proliferation, inflammation, differentiation, migration and angiogenesis, are regulated by FGFs (Prudovsky, 2021). Among the diverse recombinant FGFs that have been topically applied to heal wounds, FGF-2, FGF-1 and FGF-7 are some of the most often used (Table 1). FGF-2, also known as basic FGF (b-FGF), has been the most widely studied FGF for wound healing and is known to promote endothelial cell proliferation and angiogenesis (Prudovsky, 2021), as well as fibroblast proliferation (Baird et al., 1988). Topical application of FGF-2 has been shown to accelerate wound healing by promoting keratinocyte migration and differentiation, thus improving re-epithelialization (Hebda et al., 1990; Koike et al., 2020). As a 146-residue (18 kDa) protein, conjugation and chemical synthesis of FGF-2 still represents a challenge. However, many peptide mimetics of this growth factor have been identified (Baird et al., 1988). Of these, the FGF-2 106-120 fragment (YRSRKYSSWYVALKR) has been shown to promote fibroblast and endothelial cell proliferation, and angiogenesis, with similar potency to the full-length protein (Baird et al., 1988; Rubert Pérez et al., 2017). FGF-1, also known as acidic FGF (a-FGF), exhibits similar angiogenic activity and promotion of keratinocyte and fibroblast migration and proliferation to FGF2 (Thomas et al., 1985; Shipley et al., 1989). Some studies have suggested that its activity is more pronounced on keratinocytes compared to FGF-2 that shows higher activity for fibroblasts (Shipley et al., 1989). Topical application of FGF-1 to wounds has been shown to accelerate wound closure and re-epithelialization, but the dose must be tightly controlled (He et al., 2019; Hagerott et al., 2020). FGF-7, also known as keratinocyte growth factor (KGF), acts primarily on keratinocytes by promoting their migration and proliferation (Tsuboi et al., 1993). Its topical application accelerates wound healing by stimulating re-epithelialization and reducing scarring (Takaya et al., 2022; Kim et al., 2025). Although some FGF-1 and FGF-7 peptide mimetics have been reported (Oyama Jr. et al., 1996; Narla et al., 2022), few have been evaluated as therapeutic agents for wound healing (Choudhury et al., 2024), and none within proteinaceous matrices (Table 1).

The chemokine CXCL12, also known as stromal cell-derived factor 1 (SDF-1), has been widely studied for wound healing. This chemokine primarily promotes migration of progenitor cells, including epidermal stem cells and endothelial progenitor cells, facilitating re-epithelialization and angiogenesis (Lau and Wang, 2011; Guo et al., 2015; Janssens et al., 2018). Topical application of CXCL12 and CXCL12-containing materials has been shown to speed up wound healing by promoting angiogenesis and re-epithelialization (Sarkar et al., 2011; Vågesjö et al., 2018; Pereira et al., 2024). Although some peptide analogs of CXCL12 have been reported (Heveker et al., 1998), few have been studied in a skin wound healing context (Kim et al., 2023), and none within protein-based scaffolds.

Growth factors and their peptide mimetics can be useful tools for accelerating wound healing, but their short metabolic half-life and the highly proteolytic environment of the wound limits their efficacy. Their usage within protein matrices can therefore partially shield them from degradation and enable delayed release, thus potentiating their effects (Yamakawa and Hayashida, 2019; Nurkesh et al., 2020; Pereira et al., 2024).

### Antimicrobial agents

One of the main complications of chronic wounds is bacterial infection, which in turn impairs wound healing. Many wound healing matrices are therefore conjugated or loaded with antimicrobial agents. In this review, we will mainly focus on antimicrobial peptides (AMPs), which also elicit growth factor-like activities, but a variety of other antimicrobial molecules have been used in combination with protein matrices for wound healing.

AMPs, also called host defense peptides (HDPs), are found in most living organisms and act not only as antimicrobials but also as modulators of the host immune system. The human skin is naturally rich in AMPs, some of which have been used in wound healing scaffolds (Mangoni et al., 2016). The principal classes of human antimicrobial peptides are the cathelicidins and the defensins. The most studied cathelicidin is the 37-residue peptide LL-37, which elicits antibacterial and antiviral activity, as well as chemotactic, angiogenic and fibroblast proliferation properties (Duplantier and van Hoek, 2013). Topical application of this peptide has been shown to reduce infection and accelerate wound healing via stimulation of re-epithelialization and angiogenesis (Ramos et al., 2011; Duplantier and van Hoek, 2013). Defensins are divided in two subfamilies. The α-defensin subfamily, comprises human neutrophil peptides 1–4 (HNP1-4), and the β-defensin subfamily comprises 31 human β-defensins (HBDs), of which HBD1-4 are the most widely studied. The effects of human defensins in wound healing have been documented in multiple review articles (Steinstraesser et al., 2008; Li et al., 2022; Tan et al., 2022). Apart from their antibacterial and antiviral properties, defensins, specifically HNP1 and HBD2-3 also act on re-epithelialization by inducing keratinocyte migration and proliferation (Gursoy et al., 2013; Takahashi et al., 2021), and on angiogenesis by inducing VEGF production (Niyonsaba et al., 2007; Baroni et al., 2009; Petrov et al., 2013). They also promote fibroblast proliferation as well as collagen deposition (Oono et al., 2002).

### Functionalization strategies

One of the simplest strategies to incorporate functional moieties into a matrix is loading, a method in which the molecules are non-covalently entrapped within the hydrogel network. Hydrogel loading can be achieved by mixing therapeutic molecules with the pre-gel solution before its gelation is initiated (Chen et al., 2016; Heher et al., 2018; Pereira et al., 2024), or by adding the molecule to a preformed hydrogel and allowing it to diffuse into the matrix (Kim et al., 1992; Abdelhamed et al., 2015). Hydrogel loading is relatively simple and allows sustained release (Kim et al., 1992; He et al., 2019; Nurkesh et al., 2020). The main strategy to covalently functionalize protein matrices with bioactive moieties is genetic or synthetic conjugation, in which two proteins are expressed or synthesized as a single fusion protein incorporating a linker between the self-assembling protein and the bioactive sequence (Genové et al., 2005; Gomes et al., 2011). This strategy allows higher retention of the bioactive moiety within the matrix, although its release can be modulated by using cleavable linkers (Charati et al., 2009). Other covalent functionalization strategies include usage of chemical crosslinkers such as NHS (N-hydroxysuccinimide) esters, which react with amines of lysine residues or the N-terminus of proteins, or maleimide NHS esters, that can crosslink the thiol of cysteine residues with amines (He et al., 2011; Wohlrab et al., 2012). Such chemical crosslinking also allows functionalization of non-proteinaceous molecules but reduces control over the exact content and localization of the functional groups within the hydrogel compared to protein fusions. A slightly less common functionalization method consists of using non-covalently interacting protein domains. In this method, the protein matrix contains domains recognizable by protein-binding domains that are fused to the bioactive protein. For example, growth factors can be produced as fusions with collagen-binding domains to be added to collagen-based matrices (Long et al., 2020). The choice of functionalization strategy mostly depends on the therapeutic molecules and the properties and chemistry of the matrix to be functionalized.

## Self-assembled protein matrices

Many polypeptide sequences have the capacity to self-assemble into a diversity of tridimensional nanostructures, such as nanofilaments and nanofibers, cages and others. The self-assembly process is guided mostly by non-covalent interactions between protein monomers, including hydrophobic and ionic interactions, hydrogen bonds, and π-π stacking (Webber et al., 2016). These interactions result in the formation of quaternary supramolecular motifs, such as coiled-coils, cross-β-sheets and amphiphilic micelles (Wang et al., 2024). The same interactions allow higher-order assembly of nanostructures into matrices and hydrogels (Das and Das, 2021). Interactions involved in protein self-assembly and quaternary self-assembly motifs have been well-detailed elsewhere (Zottig et al., 2020). Self-assembling proteins conjugated with different bioactive moieties can also be co-assembled, allowing the design of multi-functionalized assemblies and matrices with a precise control over loading simply by controlling the stoichiometry of the building blocks (Bricha et al., 2023).

Polymeric matrices are of great interest to the field of wound healing because they can act as a scaffold for skin cell and for endothelial cell proliferation and as a delivery vehicle for therapeutic molecules, including the growth factors, cell-adhesion motifs and antimicrobial agents described above. Self-assembled proteins represent a scaffold of choice for wound treatment due to their biocompatibility and degradability, their porous and highly hydrated nature, their ease of functionalization, tunable mechanical properties and their ability to mimic the native ECM in the skin. In the field of wound healing, many natural and nature-inspired proteins as well as synthetic peptides have been used to engineer self-assembled materials.

## Nature-inspired protein matrices

Living organisms are an abundant source of self-assembling proteins that can be used as is or serve as inspiration for the design of protein-based biomaterials. Animal proteins as well as insect proteins have been evaluated for healing wounds. The most intuitive approach is to use self-assembly proteins found in the ECM of the skin, such as collagen and elastin.

### Collagen-based matrices

**FIGURE 2:**
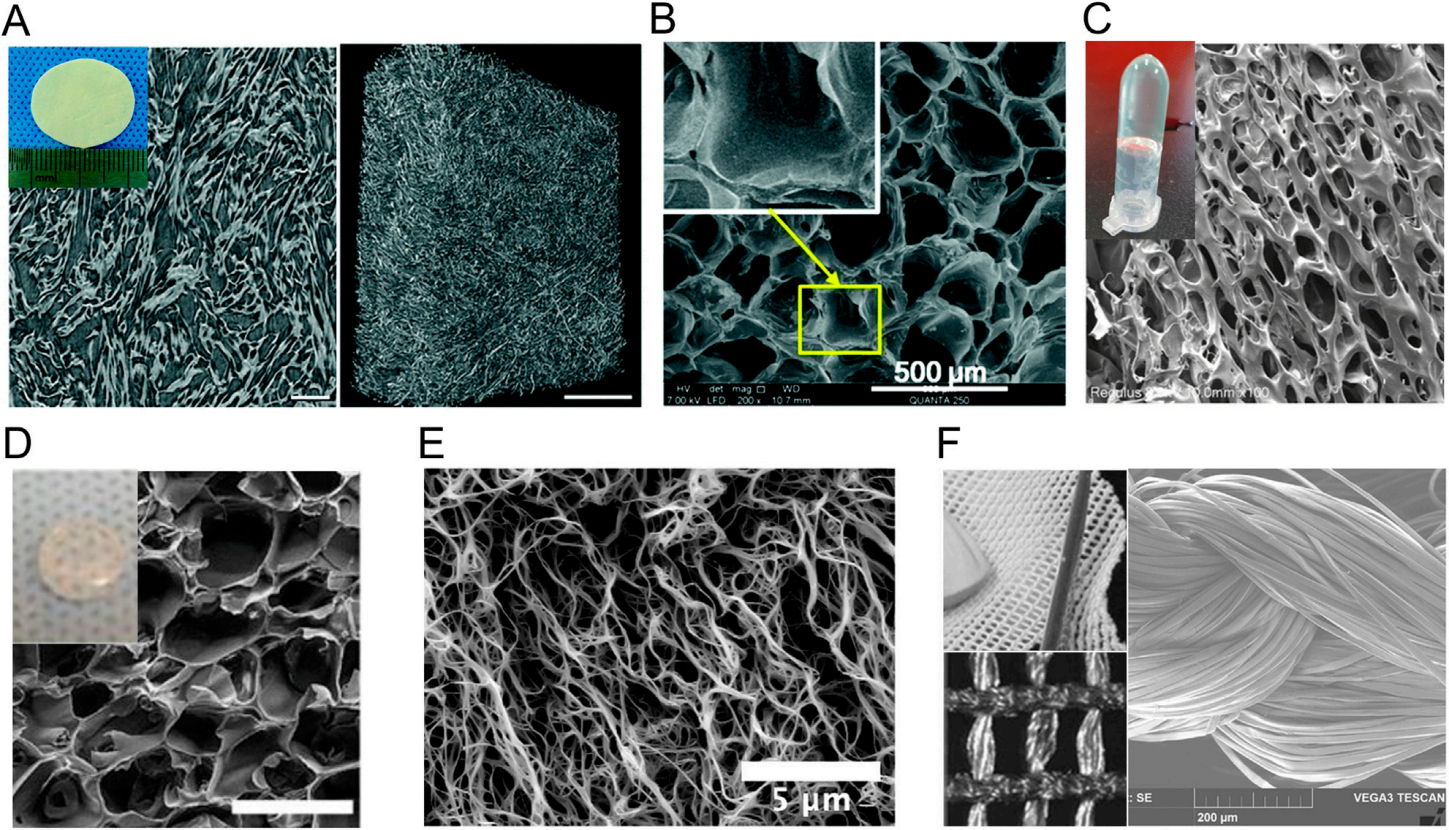
Macroscopic and microscopic appearance of nature-inspired protein biomatrices studied in a wound healing context. **(A)** Bovine collagen matrix under X-ray microscopy. **(B–F)** Scanning electron microscopy images of **(B)** gelatin-methacryloyl hydrogel, **(C)** collagen-like-peptide hydrogel, **(D)** methacrylated tropoelastin hydrogel, **(E)** fibrin-based hydrogel and **(F)** the silk fibroin surgical scaffold SERI™. Reproduced with permission from **(A)** (Long et al., 2020), **(B)** (Chen et al., 2016), **(C)** (Jia et al., 2023), **(D)** (Annabi et al., 2017), **(E)** (Pereira et al., 2023) and **(F)** (Jewell et al., 2015).

Collagen-based matrices are one of the most studied self-assembled matrices in skin wound healing. Collagen matrices usually promote wound healing by stimulation of keratinocyte and fibroblast adhesion and proliferation, thus improving re-epithelialization (De Vries et al., 1995; Middelkoop et al., 1995; Liu et al., 2021; Naomi et al., 2021; Sharma et al., 2022). Fibrillar collagens, specifically collagens I, II, III, V and XI, are structural proteins of more than 1,000 residues-length produced by fibroblasts and composing the ECM of animal organs, including the skin. These collagen molecules self-assemble and co-assemble into long fibres with a triple helix quaternary structure (Holmes et al., 2018). Most collagen-based matrices for wound healing are made from collagen I and, accessorily collagen III. A well-known example of collagen-based matrix for wound healing is Matriderm®, which has received full clearance by the FDA in 2021 (Table 2). This matrix is mainly composed of bovine skin collagens I and III mixed with 3% α-elastin hydrolysate, and has been shown to promote new collagen deposition and synthesis in the dermis (De Vries et al., 1995; Middelkoop et al., 1995). Other matrices made from bovine collagens I and III have been functionalized with angiogenic factors like VEGF and CXCL12, leading to increased blood vessel formation, ECM accumulation and re-epithelialization in diabetic and non-diabetic rats (Figure 2) (He et al., 2011; Long et al., 2020). Bovine collagen has been commonly used for such matrices, but many marine collagens have also been studied in a wound healing context (Naomi et al., 2021). For example, barramundi collagen matrices, without additional bioactive molecules, have been shown to promote wound healing in mice (Figure 2; Table 2) (Liu et al., 2021).

**TABLE 2:** Examples of nature-inspired protein matrices that have been evaluated in a wound healing context.

Scaffold protein	Bio-fabrication	Crosslinker/modulator	Added bioactive sequence	Observations and outcomes	References
Collagen-inspired					
Bovine skin collagen	Extracted	3% α-elastin	N/A	Scar tissue formation and collagen deposition in human diabetic wounds, attachment of dermal fibroblasts	De Vries et al. (1995), Middelkoop et al. (1995)
		N/A	CXCL12 and VEGF	Proliferation and migration of endothelial cells *in vitro*, angiogenesis, re-epithelialization, collagen deposition in diabetic rats	Long et al. (2020)
Barramundi skin collagen	Extracted	N/A	N/A	Proliferation and migration of fibroblasts and keratinocytes *in vitro*, angiogenesis, accelerated wound closure and re-epithelialization in mouse full-thickness wounds	Liu et al. (2021)
Collagen-like protein (CLP)	Recombinant in *E. coli*	Added Cys for disulfide bond	N/A	Adhesion, proliferation and migration of fibroblasts, collagen production, accelerated wound closure in diabetic mouse full-thickness wounds	Jia et al. (2023)
Porcine gelatin	Extracted + hydrolysed collagen	Methacrylic acid	N/A	Proliferation and differentiation of keratinocytes, proliferation of fibroblasts *in vitro*, collagen deposition, re-epithelialization and accelerated wound closure in mouse full-thickness wounds	Zhao et al. (2016), Zhao et al. (2017)
			DFO	Proliferation of fibroblasts and endothelial cells, angiogenesis *in vitro*, angiogenesis and accelerated wound closure in diabetic rat full-thickness wounds	Chen et al. (2016)
		Phenol crosslinking	IL-8 and MIP-3a cytokines	Infiltration of cells, collagen deposition, angiogenesis and accelerated wound closure in diabetic mouse full-thickness wounds	Yoon et al. (2016)
Elastin-inspired					
Tropoelastin	Recombinant in *E. coli*	Methacrylic acid	N/A	Growth and proliferation of MSCs and EPCs (angiogenesis), rapid hemostasis of rat aorta incision	Annabi et al. (2017)
α-elastin	Extracted + hydrolysed elastin	HMDI	N/A	Infiltration and proliferation of fibroblasts *in vitro*	Annabi et al. (2009)
ELRs VKVx24 + HRGD6	Recombinant in *E. coli*	Click chemistry (cyclooctyne-lysine and azidolysine)	RGD peptide (AVTGRGDSPASS)	Adhesion and proliferation of fibroblasts, myoblasts and endothelial cells *in vitro*	Testera et al. (2015), González de Torre et al. (2018)
ELR 200xVPAVG	Recombinant in *E. coli*	N/A	ABP-CM4 antimicrobial peptide	Antimicrobial activity against Gram+ and Gram- bacteria, biocompatible with fibroblasts and keratinocytes *in vitro*	da Costa et al. (2017)
Fibrin-based					
Fibrin	Mixing of fibrinogen, thrombin (and factor XIII)	Factor XIII	N/A	Migration and proliferation of keratinocytes, proliferation of fibroblasts and endothelial cells and accelerated wound closure in mouse full-thickness wounds	Pereira et al. (2023)
		Factor XIII, Heparin	FGF-2	Proliferation of fibroblasts and angiogenesis in mouse model	Jeon et al. (2005)
Silk-inspired					
Silk fibroin	Extraction from *B. mori* cocoons	N/A	N/A	Proliferation of fibroblasts, fibrin clot formation, angiogenesis, collagen deposition and accelerated wound closure in mouse wound models, re-epithelialization and wound closure in rabbit and porcine full-thickness skin wounds	Jewell et al. (2015), Kijanska et al. (2016)
Silk-sericin	Produced by fibroin-deficient *B. mori*	N/A	N/A	Proliferation and infiltration of fibroblasts and keratinocytes *in vitro*, re-epithelialization and accelerated wound closure in mouse full-thickness wounds	Li et al. (2023)
Spider silk	Collected from *N. edulis*	N/A	N/A	Angiogenesis *in vitro*, re-epithelialization, collagen deposition and accelerated wound closure in sheep full-thickness skin wounds	Liebsch et al. (2018)
eADF4(C16) artificial spidroin	Recombinant in *E. coli*	N/A	RGD peptide (GRGDSPG)	Adhesion and proliferation of fibroblasts *in vitro*	Wohlrab et al. (2012)
Resilin-inspired					
RLP12 (12x GGRPSDSYGAPGGGN)	Recombinant in *E. coli*	THPP	RGD peptide	Adhesion and spreading of hMSCs *in vitro*	Li et al. (2013)
		HMP and THPP	RGD peptide, VEGF, bFGF	Adhesion and proliferation of fibroblasts *in vitro*	Charati et al. (2009)
RZ10 (RLP)	Recombinant in *E. coli*	THP	RGD peptide	Adhesion, proliferation and spreading of hMSCs *in vitro*	Renner et al. (2012)
			VEGF-mimetic peptide KLTWQELYQLKYKGIGG	Adhesion, proliferation and spreading of hMSCs and endothelial differentiation *in vitro*	Kim and Liu (2016)

Abbreviations: VEGF, vascular endothelial growth factor; DFO, deferoxamine; IL-8, Interleukin 8; MIP-3a, Macrophage Inflammatory Protein 3a; MSCs, Mesenchymal Stem Cells; EPCs, Endothelial Progenitor Cells; HMDI, hexamethylene diisocyanate; ELR, Elastin-Like Recombinamer; FGF-2, Fibroblast Growth Factor 2; HMP, hydroxymethylphosphine; THPP [tris(hydroxymethyl)phosphino]propionic acid; THP, tris(hydroxymethyl)phosphine.

Gelatin, which consists of a hydrolysate of collagen I, has also been extensively studied for the development of wound healing matrices. Gelatin promotes wound healing through similar mechanisms as the full-length collagens, although it displays gelling properties and is more sensitive to protease degradation, owing to the fact that it is composed of short polypeptide chains of less than 20 residues (Naomi et al., 2021). Moreover, denaturation of collagen exposes naturally present cell adhesion motifs buried within the triple helical structure of collagen (Taubenberger et al., 2010), as well as functional groups for crosslinking. Crosslinking of gelatin allows control over mechanical properties and gelation of the matrix (Kang and Park, 2021), and most modern gelatin-based matrices for wound healing are therefore crosslinked. For example, gelatin is often methacrylated to gelatin-methacryloyl (GelMA) to allow photo-crosslinking *in situ*. Porcine GelMA scaffolds have been shown to promote dermal fibroblast proliferation, migration and novel collagen production, and keratinocyte differentiation and proliferation, leading to accelerated wound healing in mice models (Zhao et al., 2016; Zhao et al., 2017). Gelatin-based matrices have also been loaded with proangiogenic factors, such as IL-8, macrophage inflammatory protein 3 (MIP-3) and deferoxamine (DFO), an iron chelating agent that upregulates VEGF, resulting in improved re-vascularisation in murine diabetic wound models compared to nonloaded matrices (Figure 2; Table 2) (Chen et al., 2016; Yoon et al., 2016).

Collagens used for skin wound matrices are usually extracted from animal skin and bones that have been decellularized and defatted (Naomi et al., 2021; Matinong et al., 2022), which could decrease control over their exact composition and purity. Recombinant expression of collagen presents challenges due to its length and extensive post-translational modifications (Wang et al., 2017), which also limits the possibilities for engineering its primary structure. Bacterial collagen-like self-assembling proteins have therefore been studied as an alternative to animal collagens. Bacterial collagen-like proteins (CLPs) also assemble into a triple-helical structure and contain the signature collagen-like domains that are rich in GX_1_X_2_ repeats, where X_1_ and X_2_ are any two amino acid (An et al., 2014; Qiu et al., 2023). The *Streptococcus pyogenes* collagen-like protein 2 (Scl2) has been extensively characterized and used as an inspiration for the conception of wound healing biomaterials. The ability to recombinantly express this protein allows production of hybrid chimeric proteins containing other assembly or bioactive modules, and/or a tag for affinity purification. For example, a hybrid protein (C-eCLP3-C) containing 3 repeats of Scl-2 inspired collagen-like domains, a RGD adhesion sequence, added cysteines for crosslinking and a His tag for purification has been recombinantly expressed in *E. coli*, purified and assembled into hydrogels for wound healing (Figure 2; Table 2). Similarly to what has been reported for native collagen-based matrices, the C-eCLP3-C hydrogels promoted diabetic wound healing in mice by stimulating fibroblast migration and adhesion, and by promoting collagen expression (Jia et al., 2023).

### Elastin-based matrices

Elastin, which assembles into long elastic fibres that constitute one of the major proteinaceous assemblies of the skin ECM, has served as an inspiration for the conception of wound healing matrices. Elastin results from post-translational modifications of tropoelastin, which is a 750–800-residue protein containing highly repeated hydrophobic sequences rich in aliphatic residues (e.g., Pro, Ala, Val, Leu, Ile and Gly) (Rodríguez-Cabello et al., 2018). Abundant repetitions of the VPGVG sequence allows formation of a β-sheets secondary structure and seem to be largely responsible for self-assembly (Toonkool et al., 2001; Rodríguez-Cabello et al., 2018). Tropoelastin also contains cell adhesion motifs, including a C-terminal GRKRK that binds integrin α_V_β_3_ (Bax et al., 2009).

Since elastin is insoluble and therefore hard to process into hydrogel-like biomaterials (Daamen et al., 2001), tropoelastin is often used and crosslinked/stabilized for the conception of elastin-like assemblies and hydrogels. For example, tropoelastin has been methacrylated (MeTro) to allow photo-crosslinking *in situ* and serve as a tissue sealant. MeTro, without additional bioactive molecules, shows high adhesive properties, achieves rapid hemostasis in open wounds and supports growth and proliferation of stem cells and endothelial progenitor cells (Figure 2; Table 2) (Annabi et al., 2017). Heat-stabilized tropoelastin (HeaTro) has also been shown to promote fibroblast migration, proliferation and collagen deposition as well as neovascularization, leading to an improved wound healing process in pigs (Mithieux et al., 2018). Overall, tropoelastin-based materials have been shown to support dermal fibroblast and endothelial cell migration, attachment and proliferation (Mithieux et al., 2013).

The α-elastin is a soluble component obtained by hydrolysis of elastin (Tamburro et al., 1977) that can be crosslinked, in similar ways to tropoelastin, to obtain reticulated hydrogels. For example, an α-elastin-based matrix that was crosslinked using hexamethylene diisocyanate (HMDI) has shown potent adhesive and proliferative properties toward fibroblasts (Annabi et al., 2009).

Tropoelastin has served as an inspiration for the conception of engineered self-assembling protein fragments and short peptides called elastin-like polypeptides (ELPs) or elastin-like recombinamers (ELRs). ELRs primarily contain repeats of the elastin hydrophobic sequence VPGXG, where X can be any amino acid except proline. As previously discussed for CLPs, ELRs can also contain other self-assembling or bioactive moieties, which allow fine tuning over their mechanical and biological properties (Rodríguez-Cabello et al., 2018). Some short ELRs are chemically accessible (Aladini et al., 2016), although most of them are usually obtained through recombinant expression. ELR self-assembly is usually triggered by an increase in temperature. Some ELRs can assemble into higher order assemblies that can be electrospun into fibers, but others are engineered to assemble into other types of nanostructures, including micelles (Saha et al., 2020). Interestingly, some of these micelles have been encapsulated in fibrin hydrogels, for the conception of wound healing biomaterials, which will be discussed below (Bulutoglu et al., 2022). A combination of VKVx24, an ELR containing 24 VPGXG repeats where X is valine or lysine, and HRGD6, an ELR containing a RGD cell adhesion sequence and 20 VPGXG repeats where X is isoleucine or lysine, have been assembled and electrospun into fiber-rich materials with high potential for wound healing (Testera et al., 2015; Sousa et al., 2017; González de Torre et al., 2018). In such materials, the 2 ELRs were crosslinked using click chemistry between lysine residues functionalized with an alkyne (cyclooctyne or pentynoic acid) incorporated in VKVx24 and azidolysines incorporated in HRGD6 to stabilize fibers. These materials have not been evaluated in *in vivo* skin wound models, but have shown capacity to stimulate fibroblast, keratinocyte and endothelial cell adhesion and proliferation *in vitro* (Testera et al., 2015; Sousa et al., 2017; González de Torre et al., 2018). These observations indicate that such materials could lead to enhanced re-epithelialization and angiogenesis in skin wounds. Another ELR comprising 200 VPAVG repeats has been shown to form higher order assemblies that can be electrospun into fibres without the need for crosslinking for stabilization (Machado et al., 2009; da Costa et al., 2017). Functionalization of this ELR with an antimicrobial peptide isolated from Chinese silkworms (CM4) has allowed the formation of fiber-rich materials compatible with fibroblast and keratinocyte growth and showing antimicrobial properties against Gram-positive and Gram-negative bacteria (da Costa et al., 2017). Overall, evaluation of ELR-based matrices and hydrogels for wound healing is still at an early stage and would require further investigation.

### Fibrin-based matrices

Fibrin, just like elastin, is an elastomer, meaning it presents high elasticity. It is derived from the cleavage of the protein fibrinogen by thrombin, which exposes domains responsible for its assembly into fibers and higher-order assemblies, which are further stabilized through crosslinking by factor XIII (Kattula et al., 2017). Fibrin clots naturally play an integral role in blood coagulation and in wound hemostasis, but also in later stages of wound healing, where they serve as a provisional matrix for cells (Heher et al., 2018). Fibrin contains RGD motifs that allow adhesion and proliferation of fibroblasts and endothelial cells and is known to promote angiogenesis and re-epithelialization (Cheresh, 1987; Gailit et al., 1997). Exogenous fibrin matrices formed by mixing fibrinogen with thrombin and sometimes factor XIII have been used as surgical sealants and as delivery matrices for growth factors and other therapeutic molecules. Topical application of plain fibrin matrices has been shown to accelerate wound healing in mice by promoting keratinocytes migration and proliferation, as well as endothelial cell and fibroblast growth (Figure 2; Table 2) (Pereira et al., 2023). Fibrin matrices have also been loaded with diverse growth factors for delivery in the wound, such as VEGF (Mittermayr et al., 2008), CXCL12 (Pereira et al., 2024), FGF-1 (Pandit et al., 2000), and FGF-2 (Jeon et al., 2005), resulting in further improved re-epithelialization and/or angiogenesis. Regarding the production of fibrin gels, fibrinogen, thrombin, and factor XIII can each be extracted from plasma or recombinantly expressed and mixed in different ratios to control the mechanical properties of the resulting gels (Calcaterra et al., 2013; Heher et al., 2018). Therapeutic molecules can be added at the mixing step for loading in the resulting gel (Heher et al., 2018). Fibrin matrices are mature technologies that are FDA approved and commercially available as wound sealants under the names of Tisseel®, Beriplast®, Crosseal™ and CryoSeal® (Whelan et al., 2014). One of the main limitations of fibrin matrices is that, as an early actor in the healing process, fibrin undergoes rapid degradation in the wound environment (Whelan et al., 2014), limiting its usage in later healing stages and for chronic and hard-to-heal wounds.

### Silk-based matrices

Insects are also a great source of self-assembling proteins with interesting mechanical and biological properties for biomedical applications. One of the most used protein assemblies originating from insects is silk, which is composed of elastomeric proteins. Among silk proteins, the most studied are the ones produced by the silkworm *Bombyx mori* and by the spiders of the *Araneus* and *Nephila* genera (Chouhan and Mandal, 2020). Both silkworm and spider silk-inspired matrices have been evaluated in wound healing and demonstrated high biocompatibility and low immunogenicity in mammals (Chouhan and Mandal, 2020), despite their xenobiotic nature.

Silk fibroin (SF), which is the main component of *B. mori* silk, is known to contain GAGAGS and GAGAGY repeats that induce self-assembly into β-sheet-rich fibers (Cheng et al., 2014). Some SF-based materials, such as the SERI™ surgical scaffold, are approved by the FDA for soft tissue repair (Figure 2; Table 2) (Jewell et al., 2015; van Turnhout et al., 2018). This biocompatible and resorbable scaffold is primarily used in breast reconstruction but has also been shown to accelerate skin wound healing in mice by favoring neo-vascularization, collagen deposition, and fibroblast proliferation (Jewell et al., 2015; Kijanska et al., 2016). Other SF-based matrices have shown similar results in rabbit and porcine wound models (Zhang et al., 2017). Alternatively, silk sericin (SS), another protein constituting 25%–30% of *B. mori* silk has been evaluated in a wound healing context. SS is a glycoprotein whose aggregation into β-sheet-rich gels can be induced upon treatment with ethanol, dehydration, or crosslinking (Lamboni et al., 2015). Materials made of SS fibers exhibit antioxidant properties and support fibroblast and keratinocyte migration and proliferation, thus improving re-epithelialization and wound healing in mice (Li et al., 2023). However, SS is sensitive to denaturation and yields fragile materials, which is why most SS biomaterials also incorporate other polymers, such as alginate and chitosan (Nayak et al., 2014; Lamboni et al., 2015).

The ability of spider silk to treat wounds has also been evaluated. For example, silk directly collected from the spider *Nephila edulis* has been shown to promote angiogenesis, collagen deposition and keratinocytes and fibroblasts migration and proliferation, resulting in improved re-epithelialization in a sheep skin wound model (Table 2) (Liebsch et al., 2018). Compared to silkworm silk extraction, spider silk extraction is a cumbersome process (Chouhan and Mandal, 2020), which has prompted the development of spider silk-inspired artificial proteins that can be recombinantly expressed in bacteria. Spider silk is composed of an assembly of spidroins, which form β-sheet-rich fibers. These proteins contain repetitive cores with polyalanine repeats that are largely responsible for β-sheet formation and GGX repeats, which present a higher solubility and confer elasticity (Oktaviani et al., 2018). Artificial silks are therefore composed of a combination of polyalanine and GGX repeats. For example, a recombinant artificial silk protein, eADF4(C16), has been engineered based on 16 repetitions of the consensus sequence (GSSAAAAAAAASGPGGYGPENQGPSGPGGYGPGGP) of a spidroin from *Araneus diadematus*. Addition of a RGD cell adhesion motif to this artificial silk by maleimide crosslinking has allowed the conception of a β-sheet-rich matrix that promotes adhesion and proliferation of fibroblasts (Wohlrab et al., 2012), which is a promising feature for wound healing (Table 2). A similar β-sheet-rich matrix has been obtained by engineering an artificial spidroin containing 6 repeats inspired from the core sequence of a spidroin from *Nephila clavipes* (SGRGGLGGQGAAAAAGGAGQGGYGGLGSQGT). Genetic fusion of this protein to antimicrobial peptides HNP-2 and HNP-4 has resulted in cytocompatible matrices showing antimicrobial properties against Gram-positive and Gram-negative bacteria (Gomes et al., 2011). Nonetheless, these recombinant matrices have been less studied compared to other silk-based matrices and remain to be evaluated with *in vivo* wound models.

### Resilin-based matrices

Resilin is an elastomeric protein that self-assembles into a network of randomly coiled polypeptide chains whose tyrosine side chains are crosslinked in dityrosines and trityrosines (Weis-Fogh, 1961; Andersen, 1964). To the best of our knowledge, natural resilin assemblies have not been tested as wound healing biomaterials. However, many resilin-like peptides (RLPs) that can be recombinantly expressed have been engineered using repetitive tyrosine-containing hydrophilic domains present in resilin and resilin-like proteins. For example, RLP12 was developed using 12 repeats of the *Drosophilia melanogaster* (fruit fly) resilin motif GGRPSDSYGAPGGGN, and the RLP RZ10 was developed using 10 repeats of the *Anopheles gambiae* (mosquito) resilin motif AQTPSSQYGAP. These peptides can be crosslinked using chemical crosslinkers, such as hydroxymethylphosphine (HMP) and [tris(hydroxymethyl)phosphino]propionic acid (THPP) (Charati et al., 2009; Renner et al., 2012; Li et al., 2013; Kim and Liu, 2016). Matrices based on RZ10 or RLP12 genetically fused with a RGD adhesion motif have been shown to support adhesion and spreading of fibroblasts and mesenchymal stem cells, although these matrices have not been tested with skin cells so far (Charati et al., 2009; Renner et al., 2012; Li et al., 2013). A matrix composed of the RZ10 motif conjugated to the VEGF-mimetic peptide KLTWQELYQLKYKGIGG has been shown to promote endothelial differentiation, which leads to improved angiogenesis (Table 2) (Kim and Liu, 2016). Some composite materials containing RLPs have shown interesting properties for *in vivo* wound healing (King et al., 2019), but no materials composed entirely and exclusively of RLPs have been evaluated in this context.

### Modular and composite materials

Engineering protein mimetics (*e.g.*, CLP, ELR, artificial silk and RLP) also allows for the design of modular short peptides, such as silk-elastin-like peptides (SELPs). SELPs are most often composed of silk-fibroin-inspired (*e.g.*, GAGAGS) and elastin-inspired (*e.g.*, GVGVP) repeats and can be assembled into fibers by electrospinning (Cappello et al., 1990; Qiu et al., 2010). For example, the SELP SE-P47K has been shown to enhance fibroblast migration and collagen deposition (Ozaki et al., 2014), as well as re-epithelialization and angiogenesis of diabetic mouse wounds (Sawaragi et al., 2023). Results of phase III clinical trials in which this matrix was used were recently published and demonstrate the safety and efficacy of such matrices in acute and chronic wound healing (Sawaragi et al., 2025). SELPs are one of the most characterized modular self-assembling polypeptide sequences in the field of wound healing, but this kind of design allows the creation of a virtually infinite number of self-assembly sequences with different mechanical and biological properties.

Combining different constituents to form matrices can allow the modulation of their mechanical and biological properties. Composite materials for wound healing have been developed using protein mixtures, such as elastin-inspired proteins with collagen or gelatin (Jiménez Vázquez and San Martín Martínez, 2019), or collagen with silk fibroin (Senthil, 2024). Other composite protein-based biomaterials have incorporated glycans, such as heparin (He et al., 2019) and cellulose (Tudoroiu et al., 2023), or synthetic polymers such as polyethylene glycol (PEG) (Sargeant et al., 2012).

## Synthetic peptide matrices

As previously mentioned, extraction of natural proteins from their endogenous environment can be cumbersome, yield limited reproducibility and difficulty of characterization. These limitations can be circumvented by preparing synthetic oligopeptides. Such peptides are chemically synthesized and purified by high-pressure liquid chromatography (HPLC), ensuring high control over the purity and composition of the resulting materials. These peptides can be conjugated with short bioactive/biomimetic peptides during synthesis, and co-assembly of these functionalized peptides allow the design of multi-functionalized matrices. Furthermore, chemically synthesized peptides can easily be stored for long periods of time under lyophilized powder form before their controlled resuspension in aqueous buffer.

### β-Sheet forming peptides

**FIGURE 3:**
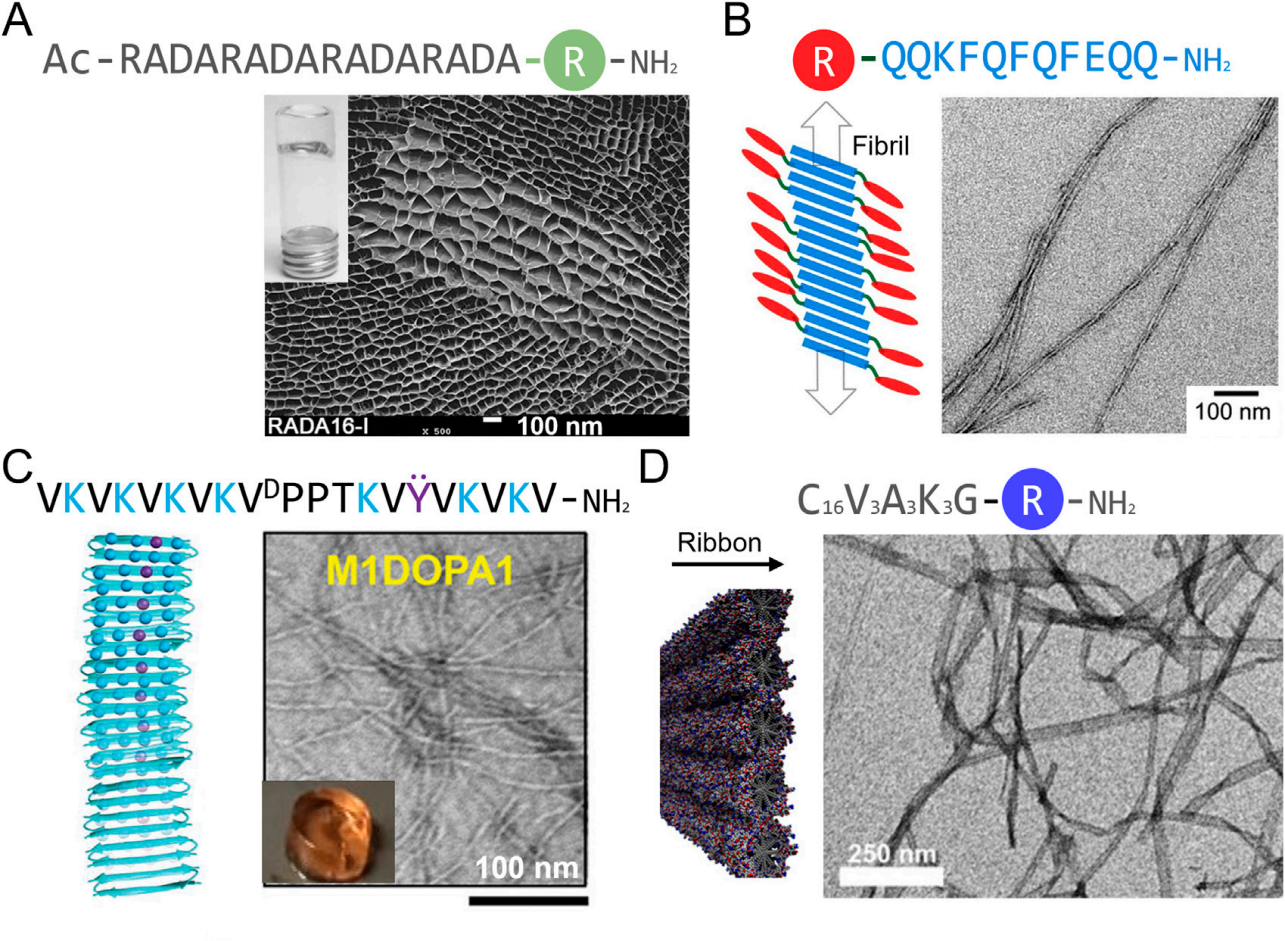
Schematic representation and macroscopic and microscopic appearance of synthetic peptide matrices studied in a wound healing context. **(A)** RADA16 hydrogel under scanning electron microscopy. **(B–D)** Transmission electron microscopy images of **(B)** Q11 peptide assemblies, **(C)** a Max1-based hydrogel and **(D)** peptide amphiphile C_16_V_3_A_3_K_3_ assemblies. R is a bioactive peptide and linker. Reproduced with permission from **(A)** (Dzierżyńska et al., 2023), **(B)** (Rudra et al., 2010), **(C)** (Fichman et al., 2021) and **(D)** (Rubert Pérez et al., 2017).

Oligopeptides assembling into β-sheet-rich nanofilaments are well studied as peptide hydrogelators. Among these, the RADA16 peptide (Ac-RADARADARADARADA-NH_2_) has been the most applied in the field of tissue regeneration (Figure 3; Table 3). This peptide is composed of four repetitions of the RADA motif (Genové et al., 2005). The alternating of hydrophobic and hydrophilic residues leads to amphiphilic stacking that allows formation of β-sheet fibers and ribbons (Zhang et al., 1993; Genové et al., 2005). This peptide has been synthesized in fusion with various cell adhesion sequences, including laminin-inspired YIGSR and RGD, allowing the design of matrices that promote endothelial cell and fibroblast adhesion and proliferation, as well as collagen deposition (Genové et al., 2005; Bradshaw et al., 2014). Co-assembly of RGD-functionalized RADA16 with a version of RADA16 functionalized with VEGF-mimicking peptide KLTWQELYQLKYKGI also favors endothelial cell migration and angiogenesis (Liu et al., 2012). RADA-based matrices that have been approved by the FDA and commercialized under the name AC5® have proved efficiency in accelerating initial wound hemostasis as well as the transition to the proliferative phase of healing during early clinical assessment (Rahmani et al., 2018; Kapp et al., 2022). RADA16 is inspired from the EAK16 self-assembling sequence (Ac-AEAEAKAKAEAEAKAK-NH_2_), in which the basic and acid residues were replaced by R and D in an effort to create RGD-like cell adhesion motifs (Zhang et al., 1993; Zhang et al., 1995). EAK is a β-sheet forming peptide found in the yeast protein zuotin that has been shown to promote rapid wound hemostasis (Table 3) (Luo et al., 2011). Among other β-sheet forming peptides that have been developed, Q11 (Ac-QQKFQFQFEQQ-NH_2_) has also been studied in a skin wound healing context (Figure 3; Table 3) (Vigneswaran et al., 2016). This peptide, which can self-assemble into fiber-rich hydrogels, has been shown to be immunocompatible when topically applied to skin wounds (Vigneswaran et al., 2016). Although non-functionalized Q11 does not appear to have a significant impact on wound healing in mice, Q11 matrices functionalized with cell adhesion sequences RGD and IKVAV have been shown to promote endothelial cell attachment and spreading *in vitro* (Jung et al., 2009), which could lead to increased angiogenesis *in vivo*. So far, this peptide has only been sparsely studied in the context of wound healing.

**TABLE 3:** Examples of self-assembling synthetic peptides that have been evaluated as matrices in a wound healing context.

Scaffold peptide	Crosslinker/modulator	Added bioactive sequence	Observations and outcomes	References
RADA16	N/A	YIGSR and RYVVLPR and TAGSCLRKFSTM adhesion sequences	Adhesion and proliferation of endothelial cells and collagen and laminin deposition *in vitro*	Genové et al. (2005)
		RGD and FPGERGVEGPGP adhesion sequences	Adhesion (RGD) and migration (FPG peptide) of keratinocytes and fibroblasts *in vitro*	Bradshaw et al. (2014)
		RGD and KLTWQELYQLKYKGI	Migration of endothelial cells, angiogenesis *in vitro*	Liu et al. (2012)
		Angiogenic sequences GHK and KGHK and immunofan	Proliferation of keratinocytes and fibroblasts *in vitro*, re-epithelialization and accelerated wound closure in mouse full-thickness wounds	Dzierżyńska et al. (2023)
EAK16	N/A	N/A	Accelerated hemostasis in rabbit liver wounds	Luo et al. (2011)
Q11	N/A	RGD and IKVAV	Adhesion, proliferation and spreading of endothelial cells *in vitro*, immunocompatible *in vivo*	Jung et al. (2009)
Max1	DOPA residue	DOPA residue	Proliferation of fibroblasts *in vitro*, antibacterial against *S. aureus* and *S. epidermidis*	Fichman et al. (2021)
PA C_16_V_3_A_3_E_3_	N/A	RGD	Proliferation of fibroblasts and endothelial cells *in vitro*, re-epithelialization and accelerated wound closure in rat wound model	Zhou et al. (2019)
PA C_16_V_3_A_3_K_3_	N/A	FGF2 peptide	Proliferation and migration of endothelial cells *in vitro*	Rubert Pérez et al. (2017)

Synthetic *de novo* peptides forming β-hairpins and assembling into hydrogels have also been developed and show promising results for wound healing applications. These peptides are based on the MAX1 peptide (VKVKVKVKV^D^PPTKVKVKVKV-NH_2_), in which the alternating valine and lysine residues act in a similar manner to RADA16 and EAK16 to form β-sheet and the middle V^D^PPT sequence induces a turn (Schneider et al., 2002). MAX1 hydrogels supports fibroblast growth (Kretsinger et al., 2005) and MAX-derived peptides in which some lysine residues were replaced by arginine (VKVKVRVKV^D^PPTKVKVRVKV-NH_2_) or DOPA residues (VKVKVRVKV^D^PPTKVKVRVΫV-NH_2_) show antimicrobial properties against Gram-positive and Gram-negative bacteria (Figure 3; Table 3) (Salick et al., 2009; Fichman et al., 2021). DOPA and arginine-containing β-hairpins hydrogels also support dermal fibroblast growth and inhibit bacterial growth *in vivo* (Fichman et al., 2021), but no wound healing studies have been conducted with these synthetic scaffolds so far.

### Alkylated peptides amphiphiles

Alkylated peptide amphiphiles (PA) are composed of a hydrophobic alkyl tail, often C16, and a head group comprising a β-sheet forming motif (e.g., VVVAAA) and a polar group of 3 lysine or 3 glutamic acid residues (Figure 3; Table 3) (Rubert Pérez et al., 2017; Zhou et al., 2019). These peptides assemble into cylindrical micelles, that are often referred to as fibers, mostly by hydrophobic interactions between the alkyl tails. These micelles are stabilized by the formation of a cross-β-sheet quaternary motif (Cui et al., 2010; Hendricks et al., 2017). C_16_V_3_A_3_E_3_ peptide amphiphiles with an added RGD motif for cell adhesion (C_16_V_3_A_3_E_3_G_5_RGDS) were able to form fiber-rich hydrogels that promoted fibroblast and endothelial cell proliferation. Application of these hydrogels in a burn wound model resulted in enhanced re-epithelialization and angiogenesis, and accelerated wound closure (Zhou et al., 2019). Peptide amphiphiles (C_16_V_3_A_3_K_3_) have also been conjugated to the FGF2-mimicking peptide YRSRKYSSWYVALKR, which resulted in assemblies promoting endothelial cell proliferation and migration, thus improving angiogenesis *in vitro* (Rubert Pérez et al., 2017). These types of assemblies show high potential in the field of wound healing.

### Short self-assembling peptides

A few di- and tripeptides that assemble into fiber-rich hydrogels have also been studied in the context of wound healing. Fmoc-FF dipeptides, that assemble into fiber-rich materials through π-π stacking, have been extensively studied (Wang et al., 2023), and have been shown to support fibroblast growth (Liebmann et al., 2007). These hydrogels can also be loaded with therapeutic agents. For example, treatment with Fmoc-FF hydrogels loaded with simvastatin, a cholesterol lowering drug, has been show to accelerate wound closure in diabetic mice (Janipour et al., 2023). The presence of the Fmoc capping group can raise concerns regarding biocompatibility of hydrogels, but uncapped FF dipeptides and ^D^LFF tripeptides have also been assembled into fiber-rich hydrogels (Conte et al., 2016; Cringoli et al., 2020). The ^D^LFF tripeptide still self-assembles when conjugated to short bioactive sequences such as the LDV cell adhesion motif, yielding a hydrogel that promotes adhesion of fibroblasts (Cringoli et al., 2020). Such short peptide-based hydrogels have only been sparsely studied for wound healing *in vivo* but show interesting properties for this application, supporting additional studies.

## Matrix crosslinking

Mechanical properties of protein matrices can be further tuned by (photo)chemical crosslinking. Crosslinking usually further stimulates gelation of the matrix and increases its stiffness and resistance to degradation. Proteins contain multiple functional groups such as amines, carboxylic acids, phenols and thiols, that can be harnessed for crosslinking. Formation of disulfide bonds between thiol groups of cysteines by creating oxidizing conditions is a simple matrix crosslinking strategy. In some instances, self-assembling protein have been engineered to incorporate additional cysteine residues, allowing a higher degree of crosslinking. This is the case for the abovementioned CLP matrix, in which gelation is mediated by cysteine crosslinking in the presence of hydrogen peroxide (Jia et al., 2023). Cysteine crosslinking is a straightforward strategy that does not require incorporation of unnatural amino acids or addition of crosslinking molecules but is limited by the number of cysteines that can be incorporated into the primary sequence of the protein. Formation of dityrosine is another matrix crosslinking strategy taking advantage of natural amino acids present in the protein. Some proteins, such as resilin naturally contain dityrosine crosslinks (Weis-Fogh, 1961; Andersen, 1964), but this type of crosslinking can also be induced *in vitro* using enzymes (e.g., tyrosinase), chemical agents such as hydrogen peroxide or photoinitiators (e.g., riboflavin) (Partlow et al., 2016).

**FIGURE 4:**
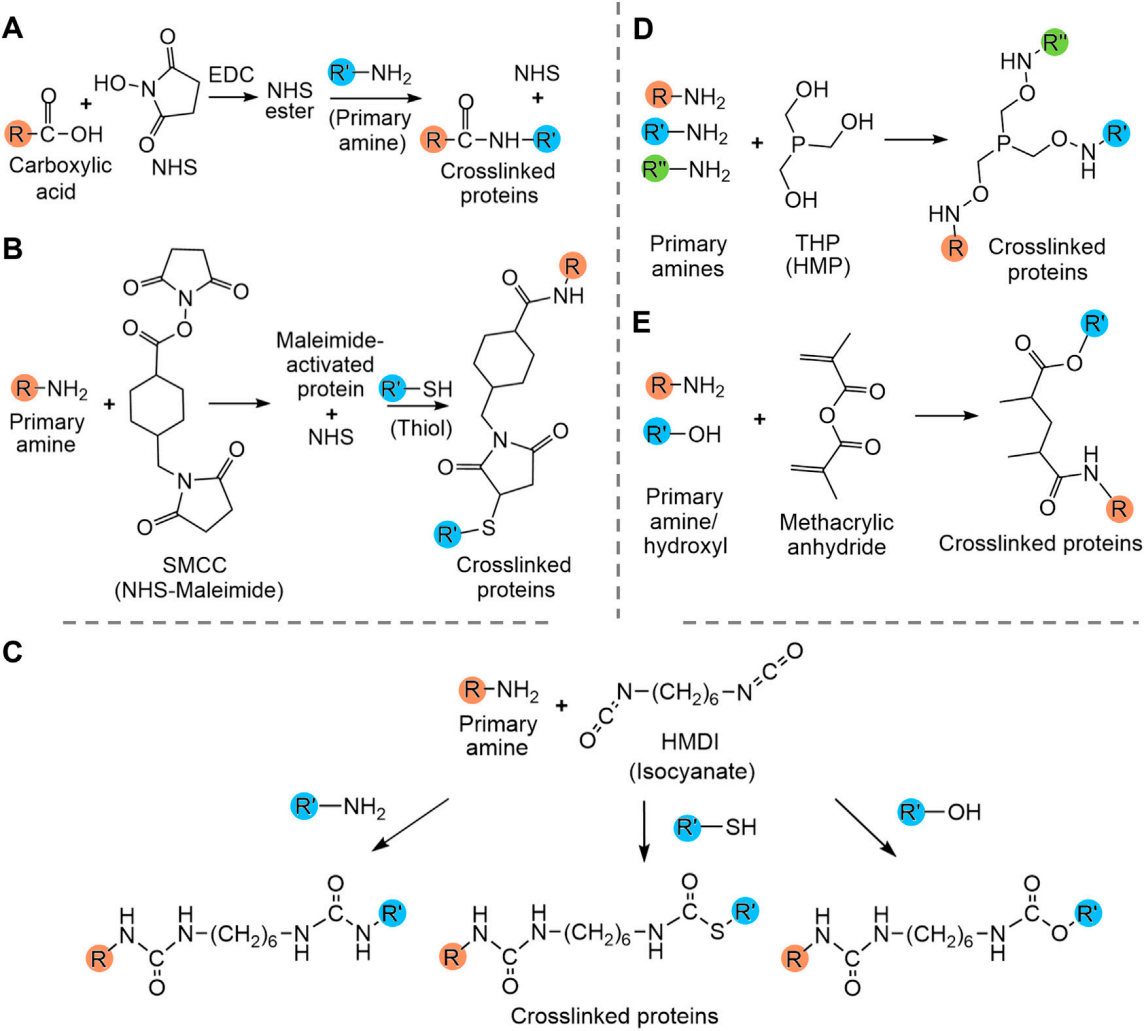
Summary of crosslinking strategies used to modulate protein hydrogel properties. **(A)** N-hydroxysuccinimide (NHS) crosslinking, **(B)** NHS-maleimide crosslinking, **(C)** isocyanate crosslinking with hexamethylene diisocyanate (HMDI), **(D)** hydroxymethylphosphines (HMP) crosslinking with tris(hydroxymethyl)phosphine (THP) and **(E)** methacryloyl crosslinking. **(E)** Amines and hydroxyls are interchangeable. R, R′ and R″ are proteins.

Crosslinking of other functional groups within the protein sequence enables a greater number of crosslinks but usually requires the addition of extrinsic crosslinking molecules such as N-hydroxysuccinimide (NHS), maleimide-NHS, hexamethylene diisocyanate (HMDI) and hydroxymethylphosphines (HMPs). These examples of crosslinkers are considered cytocompatible, which allows their usage in matrices destined for biomedical applications. NHS and maleimide-NHS esters (e.g., SMCC) are the most commonly used crosslinkers and respectively allow crosslinking between a carboxyl group and a primary amine (i.e., Lys side chain or N-terminus) and between a primary amine and a thiol groups (i.e., Cys) (Figure 4) (Hermanson, 2013a; b). They have been used for crosslinking various protein-based matrices for wound healing, including artificial silk matrices (Wohlrab et al., 2012) and bovine collagen materials, which enhanced their resistance to degradation (He et al., 2011). HMDI and other isocyanates are known to react primarily with primary amines (i.e., Lys or N-terminus) but can also react to a lesser extent with the thiol group of cysteines and hydroxy group of tyrosine (Figure 4) (Mráz and Boušková, 1999; Hermanson, 2013a). HMDI has been used for crosslinking of α-elastin-based matrices (Annabi et al., 2009). HMPs such as tris(hydroxymethyl)phosphine (THP) and [tris(hydroxymethyl)phosphino]propionic acid (THPP) also predominantly react with primary amines, which leads to crosslinking and favors hydrogelation (Figure 4) (Lim et al., 2007). These crosslinkers have been used with various proteins, including ELRs (Lim et al., 2007), and RLPs, in which the concentration of the crosslinker allows modulation of the elasticity and gel-like behavior of the matrices (Lim et al., 2007; Charati et al., 2009; Renner et al., 2012). In the case of gelatin, it is often crosslinked between primary amino groups using methacryloyl. Gelatin is first methacrylated to GelMA by reacting methacrylic anhydride (MA) with amino and hydroxyl groups of gelatin molecules (Figure 4). The extent of methacrylation can be modulated by controlling the ratio of MA to protein. Crosslinking, leading to gelation of GelMA can then be photoinduced in the presence of the photoinitiators Irgacure 2959 or lithium acylphosphinate salt (LAP) (Yue et al., 2015). This well-studied crosslinking strategy can be used to fine-tune the mechanical properties of gelatin hydrogels (Yue et al., 2015; Zhao et al., 2016; Yue et al., 2017) and has also been used in the design of tropoelastin hydrogels (Annabi et al., 2017). These chemical cross-linkages take advantage of naturally present functional groups of proteins and enable a higher extent of crosslinking within matrices than cysteine crosslinking, although such strategies often lead to a low control over the localisation of the crosslinks.

An alternative to control the sites of crosslinking is to introduce functional groups for specific crosslinking at certain positions within the polypeptide sequence. This can be done by incorporating unnatural amino acids, for example, cyclooctyne-lysines and azidolysines for bioorthogonal click chemistry (Agard et al., 2004; Sousa et al., 2017). Such modifications can be easily achieved by incorporating unnatural amino acids during chemical synthesis (Babych et al., 2024). In recombinantly expressed proteins, cyclooctyne-lysines and azidolysines can be obtained by chemical modification of lysines (González de Torre et al., 2018), but this technique does not allow precise control over the localization and extent of the crosslinking.

## Conclusion

Self-assembling peptides and proteins constitute promising molecular building blocks for the conception of materials for wound healing, because they result in highly hydrated biocompatible and biodegradable matrices and hydrogels that can mimic the ECM of the skin. These matrices can also be loaded or conjugated with bioactive proteins and molecules to induce processes associated with wound healing, such as skin cell proliferation and migration, angiogenesis and re-epithelialization. Matrices inspired from natural proteins such as collagen, elastin, fibrin, and insect proteins silk and resilin, as well as synthetic peptides have indeed shown interesting results *in vitro* and in pre-clinical trials. Some collagen-, fibrin- and SF-based matrices have even been approved for clinical usage in humans. However, their usage in clinical settings remains limited to this day. Main drawbacks of protein matrices in the field of wound healing include limited stability, unadapted mechanical properties, high cost of biofabrication and complicated purification. Extraction of natural proteins and chemical synthesis are indeed costly, but design of artificial proteins inspired from natural proteins (e.g., CLPs, ELRs, artificial silks, RLPs) allows recombinant expression, usually yielding higher quantities and easier purification than extraction processes. As for degradability and mechanical stability, crosslinking strategies and modular proteins have been developed in recent years to modulate these properties in protein hydrogels. Mixing with other natural polymers such as chitosan and cellulose, or synthetic polymers such as PEG and PLGA has also been used to modulate mechanical and biological properties of protein matrices (Sargeant et al., 2012; He et al., 2019; Kumar et al., 2023; Tudoroiu et al., 2023). Resulting hybrid materials have been more studied in recent years, showing interesting results for wound healing. The extensive studies on self-assembling protein-based matrices not only shows their potential for the field of wound healing but also represents groundwork for other recently explored approaches such as the design of modular proteins and composite matrices made of proteins and other polymers for tissue regeneration.
